# A Comparative Study of Facial Asymmetry in Philippine, Colombian, and Ethiopian Families with Nonsyndromic Cleft Lip Palate

**DOI:** 10.1155/2012/580769

**Published:** 2012-10-24

**Authors:** Liliana Otero, Luis Bermudez, Karina Lizarraga, Irene Tangco, Rocelyn Gannaban, Daniel Meles

**Affiliations:** ^1^Dentistry Faculty, Pontificia Universidad Javeriana, Carrera 7 N0. 40-00, Bogotá, Colombia; ^2^Operation Smile Inc., 6435 Tidewater Drive, Norfolk, VA 23509, USA; ^3^Operation Smile Philippines Foundation Inc., 19th Floor, UCPB Corporate Offices Building, 7907 Makati Avenue, 1209 Makati City, Philippines; ^4^Operation Smile Ethiopia, 2nd Floor Office Number 206 D, Tebaber Berta Building, Ethio-Chinese Friendship Road, P.O. Box 12129, Addis Ababa, Ethiopia

## Abstract

*Objective*. To compare the asymmetry displayed by Philippine, Colombian, and Ethiopian unaffected parents of patients with nonsyndromic cleft palate (NSCLP) and a control population. *Methods*. Facial measurements were compared between unaffected parents of NSCLP patients and those in the control group for three populations from South America, Asia, and Africa by anthropometric and photographic measurements. Fluctuating and directional asymmetries, height and width proportions, were analyzed and compared. *Results*. Fluctuating asymmetries (ear length, middle line to Zigion perpendicular for left and right sides) and variations in the facial thirds demonstrated statistical significance in the study group of unaffected parents from Colombia and Philippines, while increased interorbital distance was evident in the unaffected Ethiopian parents of NSCLP patients. *Conclusions*. The facial differences in unaffected parents could indicate an underlying genetic liability. Identification of these differences has relevance in the understanding of the etiology of NSCLP.

## 1. Introduction

Cleft lip with or without cleft palate is major structural birth defect that represents a serious public health problem. The nonsyndromic cleft lip palate (NSCLP) prevalence in Ethiopia is 1.49/1000 [1], in Philippines 1.94/1000 [2], and 1.59/1000 [3] in Colombia. This complex multifactorial disease is characterized by genetic heterogeneity, variable expression, and reduced penetrance. Although some environmental and genetics factors have been associated with the etiology of NSCLP, the phenotype definition and the ethnic influence in this pathology result in inconsistent results [4].

It has been suggested that phenotypic features play a role in the familial transmission patterns of orofacial clefts [5]. Several studies demonstrate that unaffected relatives within NSCLP families have different craniofacial features [6] suggesting that certain heritable aspects of facial form represent either a risk factor for clefts or a subclinical manifestation of this anomaly [7]. Some studies suggested that morphological variations and associated traits may represent cleft microforms or may result from more generalized development disturbances [4].

Suggested phenotype risk factors for oral clefting include midfacial deficiency, thin upper lips; increased lower facial height, interorbital distances, nasal cavity, and upper face width [8]; reduced upper face height [9], rotated mandibular position, orbicularis oris muscle defects [10], hypodontia [11], and nonright handedness [12]; asymmetrical dental development and fluctuating and directional facial asymmetry [13].

Deviations from symmetry could represent the phenotypic record of development instability [14]. Genetic and environmental factors contribute to development instability. The directional asymmetry (DA) occurs when one side of a bilateral trait is systematically larger than the other. Fluctuating asymmetry (FA) describes random deviations from symmetry in normally bilateral symmetric structures [15]. The variance in differences between the right and left sides determines the degree of FA [16]. Therefore, the presence of DA and FA suggests the breakdown of a control process in the morphogenesis.

It has been suggested that DA and FA may be a microform of NSCLP and could be a risk factor for clefting [17]. Fluctuating asymmetry in the right and left palm and in the first mandibular molars was reported for the first time in NSCLP children by Adams and Niswander [18]. Later Crawford and Sofaer [19] suggested using the familial dermatoglyphic asymmetry as an indicator of a genetic predisposition to clefting. McIntyre and Mossey [20] reported DA represented by larger horizontal values for the left side of the face and smaller vertical values for the right side of the face in unaffected parents of NSCLP patients. Fluctuating ear-length asymmetry and FA for palpebral fissure width have been recently reported in Chinese NSCLP patients and in their unaffected parents, respectively [21].

Bearing in mind the genetic heterogeneity and the variable expression of NSCLP, a study was designed to test the hypothesis that the facial phenotype in the parents of patients with NSCLP is a predisposing factor in the genesis of oral clefts in different ethnic groups. The present study sought to identify differences between the facial morphology of parents of children with NSCLP and parents of unaffected children, in Ethiopian, Colombian, and Philippine populations. 

## 2. Materials and Methods

### 2.1. Sample

The patient group included patients with nonsyndromic cleft lip with or without palate between the ages of 3 months and 13 years from Colombia, Philippines, and Ethiopia. The unaffected parents of NSCLP patients from these populations were selected. The criteria for inclusion in the study group included: (1) biological parents of NSCLP patients, (2) between the ages of 26 and 43 years, (3) from Ethiopia, Colombia, or Philippines, (4) no history of trauma, surgery of congenital defects in the face, and (5) no dental prosthesis. Parents of healthy children in the three populations represented the control group. The control group was ethnically and epidemiologically matched to study group. Criteria for inclusion of the control group included: (1) healthy parents from Ethiopia, Philippines, or Colombia, (2) between the ages of 26 and 43 years, (3) no family history of any type of orofacial clefts or congenital defect, (4) no history of trauma and surgery in the face, and (5) no dental prosthesis. Fifty-eight unaffected parents of NSCLP patients from Philippines, 67 unaffected parents from Ethiopia, and 60 unaffected parents from Colombia were chosen for this study. A total number of 51 individuals from Philippines, 70 from Ethiopia, and 50 from Colombia were included in the control group. The study and control groups from Philippines and Ethiopia were selected during medical programs conducted by Operation Smile in these countries. The control and study groups from Colombia were selected from patients who assisted at Dentistry Faculty at Pontificia Universidad Javeriana. A total of 356 individuals were evaluated (see Table 1). The study protocol was reviewed and approved by the Ethical Scientific Board at Pontificia Universidad Javeriana in Colombia and by Ethical Committees at Hospitals in Addis Ababa, Ethiopia, and in Manila, Philippines.

### 2.2. Phenotype Analysis

 Frontal photos were taken in the study and control groups. Anthropometric measures were taken with a millimetre-ruled calliper to validate the possible asymmetries found in the photos. Fluctuating and directional asymmetries, height and width proportions, were measured and compared. Frontal photographs were taken with a Sony camera DSCH5 7.1 MP, with a Carl Zeiss de 2.7–4.5/5.2–7.8 lenses from 1,20 m away from the subject.

Symmetry measurements were analyzed dividing the face vertically and horizontally. Horizontal lines adjacent to hairline, the nasal base, and menton divided the thirds. The ideal face has equal thirds. The rule of fifths was employed in each person to analyze transverse facial proportions. The face was divided into five equal parts from helix to helix of the outer ears (outer orbital width, forehead width, nasal width, zygomatic width, and maxillary width). Each of the segments should have the same width as one eye. A vertical line from the inner cantus should be coincident with the base of the nose. The line from the outer canthi of the eyes should be coincident with the gonial angles of the mandible and the commissure width should be coincident with the medial limbus of the eyes [22]. The horizontal thirds, vertical fifths, and the vertical length for different structures were measured using the Dolphin screen ruler and Vistadent software. All measures were compared in each individual by proportions. No standard measures were employed to identify the possible differences between the unaffected parents and the control group. 

#### 2.2.1. Vertical Measurements

Superior, inferior, and middle thirds; distance from internal canthal to alar base (D1); pupil to commissure (D2); ear length (D3); inferior border of ear to inferior border of mandible (D4).

#### 2.2.2. Horizontal Measurements

**Figure 1:**
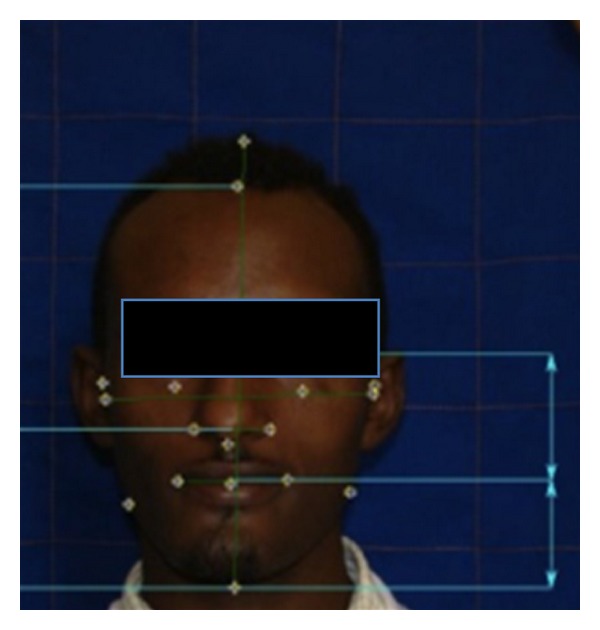
Facial measurements.

Interorbital distance, subnasal to left and right alar (D5); philtrum to left and right commissure (D6); commissure to Zigion perpendicular for left and right sides (D7). See Figure 1. 

### 2.3. Statistical Analysis

Intraobserver reliability of anthropometric and photographic measurements was analyzed by a series of paired Student's *t*-tests. Shapiro-Wilk tests were carried out to test the normality of distribution of the data in all groups. SPSS was utilized for statistical analysis of the measurements and descriptive variations. Standard *t*-tests describe differences in the means of all measures, while odd ratios (OR) were used to compare whether the probability of clefting in offspring was the same for all groups.

## 3. Results

A satisfactory level of intraobserver reliability for anthropometric and photographic measurements was proved by the fact that no statistically significant difference had been found between the duplicate measurements at a 5% level of confidence with Student's *t*-test. Comparison of measurements among the gender-matched parents of NSCLP patients and the control group showed no statistically significant difference in the three populations, however, this finding could be attributed to small sample for males in Colombia and Philippines and for females in Ethiopia (see Table 1). There was no significant difference between the type of cleft in NSCLP patients (bilateral, right/left unilateral) and the asymmetry in their parents.

Table 2 provides the *P* and OR values for the seven vertical measurements and four horizontal measurements in the three populations. 

Unaffected parents of children with NSCLP in Philippines showed diminished superior third and increased middle and inferior facial thirds compared with the parents of the control group. In Colombian parents of NSCLP patients, these measurements showed statistical significance for middle and inferior facial thirds but not for superior third. There was no statistically significant difference between facial third proportions in Ethiopian parents.

The ear length of Colombian and Philippines parents of NSCLP patients looked larger than those in the control group. No significant findings were observed in the Ethiopian population (see Table 2). Other fluctuating asymmetries found in the study group of the Philippine population but not for Colombian and Ethiopian populations were (1) distance between the inferior border of ear to inferior border of mandible, (2) distance subnasal to left and right alar, and (3) philtrum distance to left and right commissure. Fluctuating asymmetry was found in the parents of NSCLP patients in Philippine and Colombian groups for the distance from middle line to Zigion perpendicular for left and right sides. In the Ethiopian population the unique measurement that showed significant difference between the parents of NSCLP patients and control group was the interorbital distance that was found to be higher in the study group.

## 4. Discussion

Although the relationship between cleft lip and palate and facial shape in NSCLP patients and their parents has been amply documented, there are few studies that compare the facial shape in different ethnicities. The findings in the present research showed that the facial phenotype of unaffected parents of NSCLP children differed from the controls and that those differences were not the same in three populations studied. 

One difference was represented by fluctuating asymmetries that were found in Philippine and Colombian study groups but not in Ethiopian unaffected parents. The fluctuating asymmetry demonstrated for the ear length in both populations has been previously reported in Chinese individuals with NSCLP compared with their unaffected family members [12]. Although facial FA in D4, D5, D6, and D7 showed by Philippine parents of NSCLP patients and FA in D7 showed by Colombian unaffected parents had not been reported in previous studies, skeletal DA in nasomaxillary width has been found in Costa Rican parents of NSCLP patients [23]. Additionally, nasomaxillary DA in Costa Rican parents was highly correlated with the side of the defect in their offspring in left unilateral cases. This finding was reported by McIntyre and Mossey [24] reported DA in maxilla-zygomatic complex unaffected parents of NSCLP patients in Scotland and they suggested a possible association between the left DA and cleft affected side in offspring. In our study, there was a larger nasal width on the left side in 16 unaffected parents from Philippines and 10 of these had an offspring with left side cleft, but this finding was not statistically significant. These findings suggest that FA and DA may be informative risk factors for orofacial clefting in different ethnic groups. 

The Ethiopian population did not show statistical significant asymmetries but Ethiopian parents of NSCLP children demonstrated wider interorbital distance than the control group. This result coincides with the outcomes of previous studies in other populations in countries including Scotland [8], Chile [25], and Japan [26]. Additionally, both the control group and the unaffected parents in Ethiopia showed wider interalar distance and diminished superior facial third suggesting that these features seem to be related with the ethnical phenotype in African populations [27, 28] and not with clefting predisposition as has been suggested in other ethnic groups [29, 30]. In contrast, the Philippine study group did not show wider interorbital distance but this finding could be explained by the fact that it has been hypothesized that the very high incidence of clefting in Asian-derived populations could be associated with the relatively wider faces characteristic of these populations, which would result in clefting produced by the intrinsic mesenchymal deficiency during the lip and palatal embryogenesis [31].

The shorter upper face height demonstrated by Philippine unaffected parents and increased lower face height in Philippine and Colombian study groups has been reported in previous studies suggesting potential subclinical microforms or phenotypic risk markers [6, 7, 17, 27, 30]. However, all of these results must be confirmed with 3D phenotype studies and related with genetic studies in the three populations.

Facial morphology in NSCLP could explain the genetic influences on the development of clefting in the offspring and the role of morphogenes in the etiology of this pathology in different ethnic groups. Perhaps the differences found in linkage and association studies between Asian and American population studies could explain the phenotypic variations in these populations. Although more than 20 chromosome regions have been involved in NSCLP etiology, there are few reports about the genes associated with NSCLP in African populations. It is necessary to perform future research that can identify the genes responsible for FA and DA in NSCLP in the different ethnic groups and clarify if these asymmetries represent microforms of clefting or risk factors for orofacial clefting. The differences found between three populations could be attributed to genetic or environmental factors related with the ethnic origin and geographical zone. Therefore future studies must be performed to research the interactions between genetics, epigenetics, and environmental factors in these populations. 

Comparing phenotypes of unaffected parents of children with NSCLP from different ethnic groups could help us in the understanding of the genetics of NSCLP. The etiology of NSCLP has not been clarified because of inconsistent findings shown among different populations. Further studies should be directed to research the genotype-phenotype relation in different ethnic groups including microforms in the phenotype, assuring the actual number of affected individuals and identifying genes responsible for sporadic and familial NSCLP in diverse populations.

## 5. Conclusions

The results of this study demonstrated that there are differences in the facial shape of unaffected parents of children with NSCLP in three different populations in Ethiopia, Philippines, and Colombia. However, it is important to design future studies among multiple populations using the classification in multiplex and simple families, including the subphenotypes forms of NSCLP, and analyzing three-dimensional morphometric measurements with multivariate test. Currently, we are researching the association between specific phenotypes and genotypes in several populations. Further knowledge gained about the relationship between genotype-phenotype will allow for the implementation of new strategies to prevent orofacial clefting.

## Figures and Tables

**Table 1:** Parents gender distribution.

Country	Parent cleft group (male)	Parent cleft group (female)	Parent control group (male)	Parent control group (female)	Total
Ethiopia	55	12	50	20	137
Philippines	8	50	15	36	109
Colombia	17	43	14	36	110

Total	80	105	79	92	356

**Table 2:** Statistical analysis summary of the facial measurements.

Measurements	Ethiopia		Philippines		Colombia	
	Odds ratio	*P* value ****	Odds ratio	*P* value	Odds ratio	*P* value
Vertical						
Superior third: Tri-Gl (lower)	1.6	0.15	3.2	0.01*	1	0.4
Middle third: Gl-Sn (higher)	0.6	0.30	2.9	0.01*	12.75	0.001*
Inferior third: Sn-Me (higher)	0.9	0.80	7.7	0.001*	8.26	0.001*
D1 distance from internal canthal to alar base (higher)	1.5	0.19	1.7	0.15	1	0.54
D2 pupil to commissure (higher)	1	0.49	1.9	0.06	1.52	0.38
D3 ear length (higher)	1.2	0.59	5.1	0.001*	3.17	0.04*
D4 inferior border of ear to inferior border of mandible						
D4 left higher	1.3	0.42	2.8	0.03*	1	0.26
D4 right higher	0.6	0.27	5.3	0.001*	0.75	0.63
Horizontal						
Interorbital distance	2.2	0.02*	1.8	0.12	1	0.55
D5 subnasal to left and right alar (asymmetry)	0.7	0.36	5.6	0.001*	2.04	0.04
D6 philtrum to left and right commissure (asymmetry)	0.7	0.46	10.5	0.001*	2.03	0.04
D7 midle line to zigion perpendicular for left and right sides (asymmetry**)	1.13	0.83	6.0	0.01*	3.18	0.01*

*Statistically significant (*P* > 0.05).

**Asymmetry represented by right or left side higher.
